# Help-seeking for gambling problems during the COVID-19 pandemic: a qualitative study on the experiences of Norwegian help providers and their insights into gamblers

**DOI:** 10.3389/fpsyt.2026.1734445

**Published:** 2026-02-27

**Authors:** Mathilde Moldestad, David Emmanuel Smedslund Føinum, Philip Lindner, Jakob Jonsson, David Forsström, Nathan Lakew, Per Carlbring, Ståle Pallesen

**Affiliations:** 1 Norwegian Competence Centre for Gambling and Gaming Research, University of Bergen, Bergen, Norway; 2 Department of Psychosocial Science, University of Bergen, Bergen, Norway; 3 Centre for Psychiatry Research, Department of Clinical Neuroscience, Karolinska Institutet & Stockholm Health Care Services, Stockholm, Sweden; 4 Sustainable Interaction, Stockholm, Sweden; 5 Department of Psychology, Stockholm University, Stockholm, Sweden; 6 School of Psychology, Korea University, Seoul, Republic of Korea

**Keywords:** coronavirus, COVID-19, gambling, gambling support services, help providers, Norway, pandemic

## Abstract

**Introduction:**

The COVID-19 pandemic led to significant societal disruptions, including changes in gambling availability, gambling behaviours, and demand for support services. Although gambling support services play a crucial role in aiding individuals experiencing gambling-related harm, research regarding how help providers themselves perceived the pandemic’s impact on their work and callers is lacking.

**Methods:**

This qualitative study investigated Norwegian help providers’ experiences of COVID-19 and how it affected gambling and gambling-related conversations. Semi-structured interviews with thirteen help providers were analysed using thematic analysis.

**Results:**

The analysis identified five main themes: 1) Gamblers became more open, chatty, and support-seeking, 2) help providers experienced obstacles in offering support, with gamblers being reluctant to and/or impeded from receiving help, 3) COVID-19 created an opportunity to address gambling-related issues, as many became more aware of problematic behaviours amid financial hardship and reduced access to gambling platforms, 4) gambling had a heightened appeal during COVID-19, including perceptions of greater gambling availability in addition to a desire to escape as a motive to gamble, and 5) COVID-19 had minimal impact on support conversations.

**Conclusion:**

Despite appearing somewhat contradictory, the findings offer new insights into the multifaceted impact of COVID-19 on gambling and may inform future preventive and intervention efforts.

Gambling, while recreational for most, holds the potential to escalate into a maladaptive behaviour, entailing severe consequences (1–3). In Norway, various gambling support services are available to assist individuals experiencing gambling-related harms. Such support services encompass traditional helplines, as well as industry-provided support. In this study, the term “help providers” is used as a collective term encompassing employees and/or volunteers at gambling support services, including both traditional helpline workers and employees at gambling companies who provide advice and support to customers in need. These two types of help services differ in nature, yet both contribute to the field of remote and easily accessible support. Two low-threshold gambling helplines exist in Norway: Hjelpelinjen (the National Helpline) and Spillavhengighet Norge (“Gambling Addiction Norway”). Hjelpelinjen provides anonymous phone and chat services (4), whilst Spillavhengighet Norge offers self-help groups, phone consultations, individual sessions, and assistance for affected others, provided by peer workers (5). Help is also available through proactive calls initiated by gambling monopolists, Norsk Tipping and Norsk Rikstoto, where employees contact customers showcasing risky gambling behaviour, for instance, losing large amounts of money through gambling. Research has demonstrated that most recipients of such calls appreciate the initiative, in addition to proactive calls being effective in achieving more sustainable gambling behaviours (6, 7). Still, only a small proportion of those proactively contacted are referred to treatment or choose to self-exclude. Proactive calls have rather been shown to primarily encourage lower limit-setting (6). In contrast to in-person services such as general practitioners, psychologists, or crisis teams, helplines and proactive calls from gambling companies offer immediate assistance that can be accessed without prior referral and free of charge. Services of this kind remain invaluable to gamblers who may face challenges in asking for help. While support services generally are intended to deliver low-threshold assistance under ordinary circumstances, major societal disruptions may fundamentally alter both gambling behaviours and patterns of help-seeking.

In 2019, the COVID-19 pandemic emerged globally, rapidly affecting daily life and society at large. Beyond the direct physical threat of COVID-19, the pandemic had pronounced effects on mental wellbeing. For instance, in Norway, one study found the pandemic to be associated with a twofold to threefold increase in anxiety and depression symptoms in adults relative to pre-pandemic levels (8). These findings were supported by another Norwegian study indicating that a considerable proportion of the general population experienced significant psychological distress during the initial phase of the pandemic, with more than one in four surpassing the threshold for clinically significant symptoms (9). Such broader psychological effects may, in turn, have impacted gambling-related help-seeking behaviours, thereby highlighting the importance of investigating the impact of the pandemic. Furthermore, beyond its broader psychological consequences, the pandemic also introduced substantial changes to the structural availability of gambling. COVID-19 introduced social distancing measures, resulting in land-based gambling opportunities being limited and/or altered, whilst online gambling remained easily accessible. One exception to the latter was online sports betting, where the availability was periodically restricted, especially in the first wave (10). Knowledge about how social restrictions affect gambling is limited, highlighting the need for further research. Some studies have examined how pandemic-related restrictions impacted gambling behaviour. For instance, a population survey examining changes in gambling problems in Norway from 2019 to 2022 found stability in the number of active gamblers during this period, besides a stable number of problem/non-problem gamblers (11). Moreover, a decline was observed in the proportion of individuals participating in paper scratch cards, bingo at physical venues, Electronic Gaming Machines (EGMs), gambling on boats operating between Norway and other countries, online poker, foreign online casino games, horse betting, private gambling, and other forms of gambling. Simultaneously, an increase was observed in the proportion who had engaged in online scratch cards, national online casino games, number games, and the bottle recycling lottery. Although the phenomenon of gambling throughout the COVID-19 pandemic in Norway has been underexplored and despite the fact that the available literature is primarily quantitative, international research has generated further insights into changes in gambling behaviour during this period. A few studies have utilized tracking data to evaluate gambling patterns during COVID-19, indicating fewer high-risk gamblers (12) and decreased overall gambling activity during the first wave (10), alongside declining online sports wagering post-outbreak (13). However, Lindner et al. (10) also found a slight increase in online casino gambling occurring synchronously with the generally declining gambling activity, although no increase in problematic online gambling occurred. Research on clinical practices during COVID-19 further supports declining gambling activity. For instance, a Finnish study found weakened demand for clinical and supportive services due to altered gambling behaviours (14). Swedish registry data support this, demonstrating no rise in treatment uptake for gambling disorder during the pandemic’s first ten months (15). Taken together, these studies indicate that COVID-19 may have had a non-exacerbating, if not mitigating, effect on gambling problems.

However, a closer examination of various gambling activities reveals a more nuanced result. In a systematic review drawing on studies worldwide, Catalano et al. (16) reported that while COVID-19 reduced land-based gambling and sports betting, evidence indicates a shift to increased online gambling. A Chinese cross-sectional survey study supports this, finding evidence for increased web-based gambling, in addition to increased gambling among younger individuals during COVID-19 (17). A systematic review of survey data concludes that while overall land-based gambling decreased, gambling among vulnerable individuals—such as young males and problem gamblers — increased (18). Preliminary evidence from an early review suggested that during COVID-19, gambling activity either decreased or remained the same for most gamblers, whereas problem gambling increased (19). Furthermore, a Swedish study on individuals and gamblers with common mental disorders found that both pre-pandemic gamblers and pandemic-onset gamblers showed a high prevalence of at-risk and problem gambling, alongside isolation and worry correlating with higher degrees of problem gambling (20). Lastly, some narrative reviews highlight that much of the research indicating declining gambling activity relies on self-reports, with few studies utilizing tracking data (21). Collectively, these studies imply that the impact of COVID-19 may have been more complex than research initially appeared to indicate, with a possible increase in online gambling, problem gambling, and gambling among vulnerable individuals. The diverging findings signal a need for further research on the impact of COVID-19 on gambling behaviour. Furthermore, it may be argued that more nuanced knowledge could be achieved by examining the phenomenon from a novel perspective.

Importantly, the impact of COVID-19 on gambling activities extended beyond players, also influencing the support services aimed to assist them. For instance, during the pandemic, helplines adapted to social restrictions by implementing online services. Additionally, during this period, telephone-based and online help services became particularly important as restrictions limited the availability of traditional support services. However, few studies have evaluated how COVID-19 affected the use of such gambling help services. One interrupted time series (ITS) study analysed data from Sweden, Finland, and Denmark, finding inconsistent patterns in calls across the countries during the phases of COVID-19 (22). This likely reflects differing policy responses to the pandemic. Another ITS study found a substantial decrease in gambling helpline calls in Ontario, Canada, following the onset (23). However, calls from younger individuals increased (23), and younger gamblers were regarded as especially vulnerable during COVID-19 (24). Another Ontario-based ITS study indicated increasing helpline calls concerning online gambling after an initial drop in gambling-related calls, likely due to expanded gambling opportunities and heavy marketing (25). Taken together, research on helpline usage during COVID-19 is clearly scarce, and research into the specific experiences of help providers at call services in relation to COVID-19 is lacking, highlighting a major gap in current knowledge. Additionally, even though some research has examined the effects of COVID-19 on the utilization of gambling support services, those studies do not address the experiences and perspectives of the support service workers. Moreover, a few studies have explored the experiences of individuals working at non-gambling support services, such as volunteers at crisis lines and helplines (26, 27). However, those studies do not consider gambling support services specifically and do not address gambling-related issues. Collectively, these gaps in the literature highlight the demand for closer examination of how workers at gambling support services experienced the pandemic and its impact on gambling, particularly from the perspective of those providing the services.

Against this backdrop, including limited research on the help provider perspective during COVID-19 and inconsistent findings on the impact of COVID-19 on gambling and help-seeking, we conducted an exploratory study examining help providers’ experiences during the pandemic, addressing the following research question: How did gambling help providers in Norway experience the COVID-19 pandemic and its impact on gambling and gambling-related conversations? The exploratory question allowed for elucidating the complexity and nuance of participants’ experiences. The study aimed to capture the perspectives of gambling help providers in Norway during COVID-19, examining both their experiences and their insights into gambling behaviour during the pandemic. By considering the perspective of help providers, the present study addresses a major knowledge gap both nationally and internationally, offering insights that serve to complement the current literature. Additionally, investigating the effects of COVID-19 on gambling and help service utilization may have practical value, potentially informing and guiding future crises or lockdowns. Understanding the experiences of help providers may facilitate effective delivery of gambling help services during demanding societal circumstances. Moreover, the present study is situated within a Norwegian context, particularly relevant due to Norway’s well-established gambling support services - predominantly publicly funded or provided by non-profit organizations - and unique regulatory framework (28). Addressing a specific national context enabled us to thoroughly explore how pandemic-related changes were experienced within a relatively uniform service model. Though context-specific, the findings may hold relevance beyond Norway due to internationally comparable support services structures and similar pandemic-related challenges. Turning to the subjects, we found that interviewing help providers presented an opportunity to (A) capture a wide range of experiences, as each participant bore insights derived from numerous gamblers, and (B) understand how COVID-19 impacted support operations. As such, this study highlights the dual perspectives provided by each participant: the help providers’ personal experiences with offering support services during COVID-19, and their second-hand views on how gamblers experienced the pandemic, providing multiple layers of insight from the same source. Moreover, in addition to offering a novel perspective on gambling problems, by-proxy experiences add value by supplementing self-reports from gamblers. To our knowledge, this study is the first to address how help providers themselves experienced the pandemic and can thus offer a new perspective on how COVID-19 influenced the nature and development of gambling problems. Addressing a research question of both theoretical and practical significance, the present study aims to provide insights that inform practice, policy, and future research on gambling support systems and their adaptability in times of societal disruption.

## Materials and methods

### Sample and procedure

Participants were recruited through Hjelpelinjen, Spillavhengighet Norge, Norsk Tipping, and Norsk Rikstoto. Both proactive callers and workers at self-directed helplineswere included in the sample. While these two service types differ in nature, they share the common denominator of providing help and support to individuals experiencing gambling problems. As such, including both allowed us to capture a broad range of experiences. An e-mail conveying relevant information and a request to assist in the recruitment was sent to contacts within the organizations, who forwarded the invitation to relevant colleagues. Subsequently, interested parties were contacted via telephone or email to schedule the interview – all provided informed consent. To be included, participants had to be engaged in support work either (a) before and during, (b) during and after, or (c) before, during, and after COVID-19. Of the thirteen participants, all but two were classified to belong in the final category, while the remaining two (participants 3 and 7) fell into category b. Overall, contributions to the various themes appeared largely comparable across both temporal categories of participants. Participants 1–3 were trained employees at Hjelpelinjen and thus fell into the category of workers at self-directed helplines. Participants 4–6 were proactive workers from either Norsk Tipping or Norsk Rikstoto. Participants 7–13 were associated with Spillavhengighet Norge. All three men fell into this category. The sample size of thirteen was deemed sufficient to achieve thematic saturation while maintaining analytical depth, consistent with qualitative research norms (29). No participants withdrew from the study. See **Table 1** for additional sample characteristics.

**Table 1:** Sample characteristics (n = 13).

Characteristics	Category	Value
Mean age		49 years (SD = 10.98 years)
Sex	Females	10
	Males	3
Nationality	Norwegian	12
	Other	1
Organization	Spillavhengighet Norge	7
	Hjelpelinjen	3
	Norsk Tipping	2
	Norsk Rikstoto	1
Estimated number of helpline calls conducted		50 - 15 000

During spring 2024, individual phone interviews were conducted by members of the research team (MM and DESF) both with prior experience in qualitative interviewing. A semi-structured interview guide containing open questions was used to explore the perceived effects of COVID-19(e.g.,”How did you perceive the pandemic’s impact on the content of the conversations?”). The interview guide was developed to examine the experiences of help providers, and all questions were intended to elicit information pertinent to the research question, specifically participants’ accounts of how the pandemic impacted gambling and gambling-related conversations. Thus, each question was carefully formulated and operationalized to capture aspects of the research question. The first draft of the interview guide was developed by the last author, SP. Feedback on the guide was then received from authors JJ, PL and PC, whereupon the guide was revised. All authors contributing to the development of the interview guide have extensive experience within the gambling field. The iterative approach facilitated the relevance and clarity of interview questions. All questions were asked in Norwegian. The average duration of interviews was approximately 58 minutes, ranging from 24 to 98 minutes. Audio recordings were stored securely until transcribed and deleted. No repeat interviews were conducted. Participants were compensated with gift cards worth five hundred NOK (about 50 USD). Following all interviews, field notes were made, exploring emerging reflections. Due to time and resource constraints, transcriptions were not returned to participants for member checking.

### Data analysis

Transcriptions were analysed using thematic analysis (TA) (30). Data was analysed inductively (bottom-up) and semantically, meaning that themes were derived from participants’ descriptions and based on the manifest meanings of the data. A realist approach, reflecting reports of the experiences, meanings, and reality of the participants, characterized the analysis (30).

MM conducted the analysis, following Braun and Clarke’s (30) six phases: (1) familiarization, (2) coding, (3) generating initial themes, (4) reviewing themes, (5) refining, naming, and defining themes, and (6) writing the report. Familiarization was done by transcribing and reading interviews, alongside noting initial reflections. In phase two, relevant segments were provided with a meaningful description (a code). An example code was: “Symptoms or problems worsened during the pandemic”. Coding was done systematically using the NVivo 14 software. To assess inter-rater reliability (IRR), a second coder (DESF) independently applied the codebook to four random interviews. Coded parts were marked, withholding the applied code, before DESF received the codebook and transcripts. Subsequently, each code was assigned a number, before noting which code each coder used on all excerpts. The online software ReCal2 (31) calculated inter-reliability indexes, with the percent agreement measured at 93.6%, and Cohen’s Kappa at.93. Interpretation guidelines for Cohen’s Kappa suggest that scores above.90 indicate “almost perfect” agreement (32). In phase three, initial themes were generated by identifying patterns across codes. During phases four and five, themes were continuously reviewed, named, and defined. Themes were revised when overlapping, thin, and underdeveloped, and when there were contradictions within themes. SP credibility checked the final themes. The present article was written in the final phase. For presentation purposes, illustrative excerpts were translated into English and included where appropriate, with quantifiers (a few = one – three, several = four – six, many = seven – nine, and most = ten – thirteen) indicating the number of participants expressing similar experiences.

### Ethical considerations

The Regional Ethical Committee for Medical and Health Research Ethics concluded that the project fell outside the Norwegian Research Act since no health data were collected from the interviewees (ID: R3289), hence it was exempted from ethical approval.

## Results

The results are organized around five overarching themes that capture the perspectives of gambling help providers in Norway during COVID-19, particularly how the workers experienced the pandemic’s impact on gambling and gambling-related conversations. The five overarching themes reflect increased openness among gamblers to discussing mental health and gambling issues, heightened barriers to providing support, COVID-19 as a potential opportunity for addressing gambling harms, the particularly strong appeal of gambling, and a limited overall impact of the pandemic. See **Table 2** for an overview of themes and subthemes, including illustrative quotes and the number of participants contributing to each theme. The themes capture recurring patterns across the empirical evidence, reflecting areas of strong consistency in participants’ experiences and perspectives during COVID-19. Direct quotations from participants are included with the aim of vividly illustrating themes, each quote carefully selected to best exemplify the given finding.

**Table 2:** Overview of themes, subthemes, illustrative quotes, and the total number of participants contributing to each theme.

Theme	Subtheme	Illustrative quote	Total number of participants (n)
Theme 1: Increased openness and willingness to discuss mental health and gambling issues.	Seeking support.	“[ … ] [The callers] were more [ … ] personal. They wanted to talk to a person. They felt they needed someone to talk to, a lot more than before.” (*Participant 13*)	12 (10 workers at self-directed helplines, 2 proactive workers)
	Attitudes of openness toward mental health matters.	“People were more open about saying, ‘this might be a difficult time. This can be hard for many’. There was more conversation about how people were doing mentally during the pandemic. It became more accepted to say, ‘I think this is tough’”. (*Participant 1*)	
Theme 2: Growing obstacles to offering support.	Reservation to receive help.	“Many people did not answer [our calls]. [ … ] I think- but I think- I think- yes, like I said, more people did not answer” (*Participant 4*)	10 (8 workers at self-directed helplines, 2 proactive workers)
	Impeded from obtaining help.	“Many people said, sort of: [*imitates whispering voice*] ‘Yes, just hold on a second, I’m just going to go into another room’, right? ‘Because my wife is sitting here’. And they sort of whispered, right, not to expose the fact that they were gambling” (*Participant 5*)	
Theme 3: An opportunity to address gambling issues	Augmented awareness of issues affecting oneself or a significant other.	“During COVID-19, some realised that ‘maybe I have gambled too much in the past’. And that it was more visible when there was nothing left to gamble on” (*Participant 1*)	13 (10 workers at self-directed helplines, 3 proactive workers)
	Gambling-reducing factors: Financial decline, and limited access.	“Those who spent less, it might be because they felt they got- had less money to spend. That they were furloughed” (*Participant 7*)	
Theme 4: Gambling had a particularly enticing appeal.	Gambling was subject to perceptions of greater availability.	“We experienced that people with gambling issues were targeted. Meaning, they received calls and text messages saying: ‘Remember to support us’, over messages such as: you as a player are a saving angel, because you save lives by visiting us and contributing. They pushed the noble purpose exceptionally much. [ … ] they were visible in an entirely different manner, because they … yeah, they sought to collect money” (*Participant 9*)	13 (10 workers at self-directed helplines, 3 proactive workers)
	The desire for escape.	“[ … ] And then they didn’t handle the situation, and gambling was their escape” (*Participant 11*)	
Theme 5: The impact of the pandemic was minimal.	The conversations were largely unaffected.	“I can’t say there was any difference (in the conversations). At all” (*Participant 6*)	3 (2 workers at self-directed helplines, 1 proactive worker)
	The gamblers remained predominantly unchanged.	“No, there weren’t any significant changes [in the gambling behaviour or problems of the callers]” (*Participant 3*)	

Due to the intertwined nature of help providers’ own experiences and their observations of gamblers, the two perspectives (participants’ first-hand experiences vs. by-proxy experiences) are presented together, rather than in separate categories. This allows for a more nuanced and authentic representation, reflecting the naturally intertwined nature of these perspectives in everyday practice, avoiding artificial distinctions not grounded in the data. Similarly, results from both types of help providers - proactive workers and workers at self-directed helplines- are reported together, with any notable differences between service types or organizations explicitly highlighted. Unless otherwise stated, both proactive callers and workers at self-directed helplines contributed similarly to themes.

### Increased openness and willingness to discuss mental health and gambling issues

This theme illustrated participants’ experiences of increased openness in conversations with callers during COVID-19. Two subthemes were identified: “Seeking support” and “Attitudes of openness toward mental health matters”.

#### Seeking support

A topic regularly emerging in interviews was the increased need for interaction and support during COVID-19. Both gamblers and affected others seemingly contacted helplines more, expressed increased need for support, and/or were more positive towards proactive calls. Participant 13 described:

“[ … ] [The callers] were more [ … ] personal. They wanted to talk to a person. They felt they needed someone to talk to, a lot more than before. Because then … then they generally knew what they would ask about. Now [during COVID-19] they called primarily to have someone to talk to”.

This indicates a shift mid-pandemic: callers became more accessible and open to conversation. Additionally, conversations transitioned from mainly involving concrete questions and advice to emphasizing interaction with an understanding listener, highlighting how support and connection gained importance during COVID-19.

Simultaneously, many helpline workers reported increasing calls. This was especially apparent for participants from Spillavhengighet Norge, who all but one described rising calls amidst the outbreak. The rise was attributed to increased organisation-visibility, a strategy employed immediately after the outbreak. Imbalanced helpline demands led to participants from Spillavhengighet Norge experiencing disbelief in their rising inquiries, as illustrated by Participant 8:

“[ … ] We [Spillavhengighet Norge] have felt so alone after the pandemic, because we received such a large number of inquiries, while other [support organizations] experienced the opposite. So, at a- there was actually a time where you felt that: are they thinking we’re not telling the truth?”.

The excerpt illustrates how the surge in help-seeking reached a level that was almost beyond belief. Moreover, a few noted longer-lasting calls, signalling increased need for support, chattier gamblers, and possibly more subjects to discuss. Additionally, a few mentioned rising inquiries from relapsing gamblers. Together, this indicated heightened demand for support.

Others saw the rising support-seeking differently. Participant 5, a proactive caller, highlighted the excess time disposable during COVID-19:

“They had a lot more time to talk. Maybe [they were] not as stressed as [they are] when we reach out to customers now. Many people said that: ‘yeah, I’m just sitting here at home’, right”.

Wanting to pass the time, gamblers may have been more willing to talk.

A few helpline workers noted rising inquiries from affected others compared to pre-outbreak, a trend they perceived as persistent. This indicates that the effects of COVID-19 extended beyond the players themselves. Similarly, many participants found that the number of calls remained high, or even continued to grow, after the pandemic. A few participants suggested that this could be related to a delayed onset of problems, where gambling issues first surfaced later due to, for instance, orientation towards other pressing pandemic-related concerns or delays in recognising or acknowledging gambling problems.

#### Attitudes of openness toward mental health matters

Another salient subject in interviews was increased openness towards mental health matters, as illustrated by Participant 1:

“People were more open about saying, ‘this might be a difficult time. This can be hard for many’. There was more conversation about how people were doing mentally during the pandemic. It became more accepted to say, ‘I think this is tough’”.

A clear focus on mental health is illustrated here, an experience shared by many. A few attributed this to increased focus and acceptance of mental health during COVID-19, as reflected in the media. Additionally, many agreed that this effect has lingered post-pandemic.

Often, and especially during the first wave of the pandemic, conversations with gamblers centred on anxiety, despair, and worry. As indicated by Participant 8, conversations were “darker”, “heavier”, and “gloomier, “changing overnight” after the outbreak, possibly reflecting how gambling-related stress increased in light of the pandemic. Additionally, conversations frequently turned to suicidality, another effect prevailing post-pandemic, according to many.

Together, the theme, including the two subthemes, demonstrates a change in the need for support mid-pandemic, thus showcasing COVID-19’s effect on gamblers and gambling-related conversations.

### Growing obstacles to offering support

Alongside increased support-seeking, difficulties in offering support were reported across the empirical evidence. This theme captured such obstacles, with the subthemes “Reservation to receive help” and “Impeded from obtaining help”.

#### Reservation to receive help

A key barrier in offering help was a perceived reluctance amongst gamblers, with some rejecting support. This was illustrated through, for instance, difficulties in reaching the gamblers. Participant 4, a proactive caller, described:

“Many people did not answer [our calls]. And more people had to call us back. So that was something that changed [during the pandemic]. I think- but I think- I think- yes, like I said, more people did not answer”

The excerpt demonstrates how the availability of customers sank during the pandemic. This phenomenon partly extended beyond proactive callers. A few inbound helpline workers described fewer inquiries during the first wave of the pandemic. Unlike helpline workers at Spillavhengighet Norge, who saw increased demand, all participants from Hjelpelinjen described a decline following the onset, where fewer people sought help. Still, this decline appeared limited to the first wave of the pandemic, likely reflecting an early transition phase.

#### Impeded from obtaining help

Besides declining support, some gamblers faced barriers to accessing help. One obstacle mentioned by several was family proximity. Remote work and homeschooling made private calls harder to conduct. Concern about being overheard prevented some gamblers from speaking openly or responding to duty of care calls. Additionally, concealing issues made conversations even more challenging to conduct, as illustrated by Participant 5:

“Many people said, sort of: [*imitates whispering voice*] ‘Yes, just hold on a second, I’m just going to go into another room’, right? ‘Because my wife is sitting here’. And they sort of whispered, right, not to expose the fact that they were gambling”.

The excerpt highlights how COVID-19 altered routines - reducing access to privacy and rendering gamblers more susceptible to feelings of shame and secretive actions. Furthermore, the strained healthcare system was highlighted as another barrier. Gamblers appeared hesitant to seek help, especially from their General Practitioner.

“Maybe one didn’t feel sick enough struggling with gambling problems, in contrast to people who were very sick due to COVID-19.”(Participant 12)

Similarly, Participant 11 emphasised a lack of capacity and being down-prioritised:

“A lot of people who contacted us were not able to get in touch with their General Practitioner, because the GPs were so busy with COVID-19, one might say. That their [the gamblers] treatment was postponed during all of this”.

These excerpts suggest that during the pandemic, gamblers experienced being sidelined and overlooked, potentially leading to serious consequences such as exacerbation of problems. Furthermore, lower-threshold services faced adaptation, possibly impeding some from receiving help. For example, distancing mandates eliminated in-person conversations. Additionally, according to some, digitalised services may have hindered people with phone anxiety, those not fond of the format, people worried that family might overhear them, and people with limited digital skills. Lastly, according to Participant 8,

“Many people found it scary to sit and talk-to talk about what one finds hard and difficult with others on-screen, so … Without receiving eye contact”,

illustrating how digitalised support services may have appeared less accessible.

Collectively, these findings demonstrate the difficulties help providers experienced in offering support during the COVID-19 pandemic, with some gamblers appearing reluctant to accept help, while others found it harder to get the support they needed. Taken together, the theme illustrates how help providers experienced the impact of the pandemic on gamblers and gambling-related conversations.

### An opportunity to address gambling issues

This third theme reflected how, according to participants, COVID-19 presented gamblers with an opportunity to address gambling issues, with the subthemes “Augmented awareness of issues affecting oneself or a significant other” and “Gambling-reducing factors: Financial decline, and limited access”.

#### Augmented awareness of issues affecting oneself or a significant other

According to several participants, recognising gambling issues was easier during COVID-19. Social restrictions provided more time for reflection, hindered concealment of problematic gambling, and increased the visibility of one’s gambling problems. Participant 1 proposed:

“During COVID-19, some realised that ‘maybe I have gambled too much in the past’. And that it was more visible when there was nothing left to gamble on”.

This illustrates how the reduced accessibility of gambling could serve as a wake-up call, making one aware of one’s own habits. Likewise, significant others were prone to discover problems:

“I think, first of all, that next of kin were a lot more attentive to what it was. I remember that I first spoke to someone during the pandemic who thought that their partner had been cheating on them. And then it turned out that [the partner] was cheating with gambling. So, I believe we had more next of kin who started contacting us because they discovered the gambling”. (*Participant 4*)

The excerpt demonstrates how the enhanced awareness - due to the unique conditions of the pandemic – also applied to others than gamblers themselves. A few participants ascribed the enhanced awareness to increased attention to household spending during financial difficulties. Accordingly, some discovered that more money was being spent on gambling than expected. Increased awareness motivated gamblers to address their problems, as Participant 1 described:

“[ … ] They [the gamblers] managed to see that ‘I now have the opportunity to do something about it [the gambling problems]. I now see that I have displayed a [gambling] pattern that is not perfectly fine”.

This excerpt indicates that for some, the pandemic offered a promising opportunity to implement changes.

#### Gambling-reducing factors: financial decline, and limited access

Several participants highlighted financial decline, including rising unemployment, as a reason to address gambling issues. Participant 7 described:

“Those who spent less [on gambling], it might be because they felt they got- had less money to spend. That they were furloughed”.

The excerpt indicates that finances are key when considering reducing one’s gambling activity. Several participants described cautious spending among gamblers, like Participant 5:

“The uncertainty that existed, it made many people more restrictive in terms of gambling. Right – you didn’t know how the society was going to develop”.

Thus, worsening finances due to the pandemic may have pushed gamblers into action.

Nevertheless, some resumed gambling once their finances stabilized, as Participant 3 explained:

“Some said that the pandemic made them take a break and that they had rather gambled before, and then because of an uncertain financial situation they took a break, and then they resumed gambling again afterward”.

This suggests that finances were not necessarily associated with a sustained reduction in gambling activity.

Moreover, some participants perceived gambling availability to have decreased during COVID-19, with reduced income or financial instability making gambling appear less accessible. Additionally, offline gambling options were closed, reducing the availability further. According to several participants, many land-based gamblers found this enforced break relieving, presenting an opportunity to establish healthier routines. Participant 2 explained:

“I think that when land-based gambling closed, you were forced to take a break, and for some this was enough to acknowledge that: ‘This is something I have to stop doing’. And they were sort of forced to find other hobbies. Or suddenly they experienced how nice it felt to actually retain the money they previously had spent on gambling”.

This illustrates how lockdown helped gamblers recognise gambling’s effect while revealing alternative activities. This was further supported by Participant 5:

“A typical sport bettor, right, who lives and breathes soccer, right, suddenly there wasn’t anything left to gamble on. And I remember some saying: ‘I have started working out a bit’. Instead of betting on soccer. [ … ] One [ … ] said: ‘Actually- My head is finally catching a break. Because I cannot gamble on anything, so I have been grabbing my trainers and stuff. Now, two hours are freed mid-day, where we can venture outside, and then I’m out running’. [ … ] Gambling was sort of [ … ] de-prioritized in the lives of some people”.

The excerpt demonstrates that, for some individuals, closing the door to gambling may have opened other doors. Moreover, missed gambling opportunities due to remote work might have decreased the perceived availability, as Participant 1 proposed:

“It was trickier to [gamble] where you used to. So if you [usually] gambled on your way to or from work, or in a break at work, then it wasn’t as convenient [anymore]”.

This suggests that pandemic-induced changes in routines hindered the continuation of one’s usual gambling behaviours, thus reducing the perceived availability of gambling. Additionally, many addressed family proximity, proposing that it impeded secretive gambling. Participant 4 explained:

“Others reduced their gambling. Because they realised that: ‘OK. This is silly – being secretive and hiding in the bathroom to gamble. I don’t want that. I’ve been confronted with why I’m constantly running to the bathroom”.

This excerpt illustrates, from another angle, how pandemic-related changes in routines contributed to the perceived reduction in gambling availability. Accordingly, reduced accessibility may have allowed gamblers to tackle their issues. Nevertheless, some returned to old habits when possible.

The preceding findings demonstrate how both pandemic-induced changes in awareness of gambling problems and gambling-reducing factors created a perceived opportunity for individuals to address their issues, thereby informing how help providers experienced gamblers and gambling behaviours amid the pandemic.

### Gambling had a particularly enticing appeal

This theme captured gambling’s appeal during COVID-19, encouraging more frequent gambling and/or higher bets. Many participants noted worsening gambling problems or symptoms. As Participant 9 explained, the desire to gamble often intensified:

“Sometimes, gambling addicts are known to gamble on everything from, yeah, tennis from unknown countries, just to have something to gamble on, because it runs at night. But now [during COVID-19] we were talking about Faroese soccer and- and Serbian women’s handball at the bottom of the division, and …”

The excerpt illustrates how gambling became especially compelling for some individuals during the pandemic. Furthermore, several participants observed increased relapses among gamblers previously in control, more new players, and more low-risk players transitioning into problematic patterns, all indicating heightened appeal. Moreover, several participants observed shifting player demographics, with a rise in younger gamblers. Additionally, a few noted rising debts, indicating heightened gambling activity. This emphasizes gambling’s strong appeal during COVID-19, explained by the subthemes: “Gambling was subject to perceptions of greater availability” and “The desire for escape”.

#### Gambling was subject to perceptions of greater availability

Most participants mentioned that gambling appeared more accessible during COVID-19. Several described amplified marketing campaigns, sometimes bordering on what participants considered unethical practices, as illustrated by Participant 9:

“We experienced that people with gambling issues were targeted. Meaning, they received calls and text messages saying: ‘Remember to support us’, over messages such as: You as a player are a saving angel, because you save lives by visiting us and contributing. They pushed the noble purpose exceptionally much. [ … ] they were visible in an entirely different manner, because they … yeah, they sought to collect money”

The quote illustrates how promotional messages issued by betting operators strongly contributed to the sense of increased access to gambling. The increased availability was also attributed to the introduction of online gambling. Many participants noted transitions to alternative gambling prospects if the preferred one was closed, for instance, land-based gamblers shifting to online venues. Participant 11 described:

“Bingo players struggled when the bingo venues closed. The problem was that they then discovered the internet, so to speak. And [they] found the worst games online. And if to speak of what’s worse or not worse, I would claim that rapid games online are somewhat worse than [games in] a bingo venue. Because then you’re at least amongst people. Considering online [gambling], you’re both alone with the problem in the first place, and the games and the consequences afterwards. So, it … I mean, some stopped [gambling] because the bingo venue closed, but I think more [people] I talked to turned more toward online gambling instead”.

This illustrates how transitions to online gambling were prominent and possibly harmful. Furthermore, participants noted that access to unregulated online gambling and the convenience of home-based gambling may have contributed to the perceived availability.

Another key contributing factor was finances. For some, social restrictions lessened non-essential spending, resulting in more disposable income. As Participant 2 proposed, excess money increased gambling:

“It was harder not to gamble because you had money in your bank account. Because you had fewer expenses than before”.

The excerpt indicates that more disposable income enhanced the temptation to gamble, thus increasing its perceived availability. In contrast, those experiencing financial instability may have gambled hoping to earn money, as Participant 7 speculated:

“Those who spent less probably did so because they had less money to spend. [ … ] But the same applies to those who gambled more; they also experienced financial hardship. And they gambled more because they wanted more money”.

This demonstrates how altered finances seemingly increased gambling’s perceived availability, according to participants.

Lastly, several participants noted altered routines mid-pandemic, increasing gambling’s accessibility. Remote work could, for instance, inspire gambling during work hours. Others explained that altered routines facilitated relapse.

#### The desire for escape

The desire to escape similarly contributed to gambling’s heightened appeal, according to participants. The hardships of COVID-19 left people needing a respite. Participant 11 explained:

“[ … ] And then they didn’t handle the situation, and gambling was their escape”.

The quote demonstrates how pandemic-related difficulties prompted individuals to escape into gambling. Using marriage as an analogy, Participant 10 explained how gambling offered distraction:

“All marriages are complicated in their way. [ … ] And if you experience troubles, at least if you are addicted to gambling in the first place, then it’s very easy to resort to online games [ … ]. You’re sort of there to not think about everything painful around you. If you’re experiencing marital problems, you back away, right? And it [gambling] becomes a sanctuary. No one’s fighting there, and no one’s demanding anything of you, besides having sufficient funds on your card, so to speak”.

As explained by participant 10, gambling offered sanctuary from one’s problems. All participants agreed that during the pandemic, many gambled to pass the time and escape boredom. Participant 11 described a gambler whose issues worsened due to the boredom of the pandemic:

“One caller had abstained from gambling for four years when everything closed. He was left sitting at home, alone, without any leisure activities to distract him, which made him pity himself. And then he relapsed, and just kept gambling”.

This illustrates how pandemic-induced boredom and the absence of daily routines prompted individuals to escape into gambling. Similarly, adrenaline-filled activities grew in temptation, with gambling being a prime example. Participant 3 elaborated:

“Life became monotone with one not being able to engage in activities and such, so they probably sought excitement and adrenaline and such, in gambling. Because they didn’t get to partake in social happenings, and so on. So, they described it as wanting something more in everyday life, and then they started gambling. Because of that”.

The quote indicates that the pandemic deprived enrichment from people’s lives, such as feelings of thrill and the experience of truly being alive. Gambling provided a legitimate alternative under strict restrictions, offering back a sense of control. Furthermore, most participants reported rising loneliness during COVID-19, another factor one might wish to escape. Gambling became an (often unconscious) escape strategy, salient in participants’ explanations of relapse or deterioration. Participant 12 described:

“I noticed people mentioning loneliness, that they were very isolated. And were sitting a lot [at home], and it was sort of one … one of the reasons people chose to gamble more, because they were bored in their own company. Or they were lonely and … and such”.

Others wanted to escape underlying problems, including mental illnesses. This was illustrated by Participant 4, who furthermore proposed that such challenges worsened due to COVID-19:

“What we discover, typically related to gambling problems, is highly complex. You might not just have or have had problems with gambling. There might be- maybe you have struggled with alcohol or drugs or other stuff previously, and/or mental health issues, especially related to anxiety and depression. And all this worsened during COVID-19”.

This indicates that the pandemic not only encouraged attempts at escapism, but also exacerbated the very problems gamblers initially sought to escape. Furthermore, solitude and privacy in a time when families were confined in close quarters might have motivated escape gambling, as Participant 2 suggested:

“Your family is gathered on week three. You need a little … Just to get away”.

Additionally, gambling posed a distraction from the pandemic, providing comfort and escape. Moreover, positivity was desired, as indicated by Participant 12:

“Maybe one needed a ray of hope. And for many, gambling is something positive and fun”.

Thus, gambling became an escape strategy to lessen the hardships experienced.

Collectively, this theme demonstrates the appeal of gambling during COVID-19, reflected in both its enhanced availability and its function as an avenue for escapism, thereby informing how help providers experienced COVID-19 and its effect on gambling and gamblers.

### The impact of the pandemic was minimal

The last theme explores COVID-19’s direct effect, or lack thereof, on support operations. This theme revolves around participants perceiving COVID-19’s impact as minimal, with the subthemes, “The conversations were largely unaffected” and “The gamblers remained predominantly unchanged”. Because less salient than other themes, it was considered a minor theme. Nevertheless, it remains an important perspective to consider.

#### The conversations were largely unaffected

A few participants found mid-pandemic conversations resembled those prior: similar topics emerged, and COVID-19 was rarely addressed. Participant 6 stated:

“I can’t say there was any difference [in conversations]. At all”.

They continued:

“I haven’t had any conversations where that [COVID-19] has been a topic. I would remember”.

This suggests minimal pandemic-related impact on conversations. Another participant claimed no rise in inquiries amid COVID-19, also indicating minimal impact.

#### The gamblers remained predominantly unchanged

A few participants perceived gamblers as largely unaffected by COVID-19, indicating no changes in behaviours or problems. Moreover, Participant 6, a proactive caller, described no rise in gambling problem severity during COVID-19, also suggesting minimal pandemic-associated impact:

“We offered the same gambling options all the way. Meaning, we weren’t closed. We didn’t have a- we offered games via Sweden anyway. So, for our clients, the [gambling] opportunities were well-known and constant, and I didn’t experience any flourishing of high-risk- the number of high-risk customers, for instance, during the pandemic.”

Taken together, this final theme demonstrates perceptions of minimal pandemic-related impact.

## Discussion

The present study explored how Norwegian help providers experienced COVID-19’s impact on gambling and gambling-related conversations, the results revealing varying experiences in both help-seeking behaviours and gambling habits. Across themes, participants described both increases and decreases in the use of support services, in addition to perceptions of COVID-19 as a window of opportunity versus a triggering context for gamblers. Lastly, a few participants suggested that the pandemic’s impact was minimal, contrasting with all other perceptions. The findings showcased the naturally intertwined dual perspectives offered by the participants, reflecting both participants’ own experiences of delivering support and their second-hand views on gamblers during the COVID-19 pandemic. First-hand experiences included differing views on the ease of support service delivery during COVID-19, contrasting perspectives on the demand for support services, and the minority perception of support provision as largely unaffected by the pandemic. By-proxy experiences, on the other hand, included perceptions of varied help-seeking patterns in gamblers, differences in gambling activity during the pandemic, and the minority perception of gamblers as largely unchanged by the COVID-19 pandemic.

Theme one suggested increased support-seeking during COVID-19. This notion is supported by statistics from the Norwegian Gambling Authority and Spillavhengighet Norge. Numbers from Spillavhengighet Norge depicted a near doubling of initial inquiries in 2020, with consistently high numbers throughout 2021 (33). Annual reports from the Norwegian Gambling Authority showed a relatively consistent number of inquiries to Hjelplinjen during the years before COVID-19 (excluding 2017, where a small jump was depicted) (34), with a slight, gradual increase in the years following (35).Althoughboth helplines experienced increasing demand, the variations in customer contact could be explained by the increased visibility of Spillavhengighet Norge.Increased openness and support-seeking, as indicated by the theme, might be related to the loneliness experienced by many during COVID-19, where merely talking to another individual was craved. As COVID-19 increased social isolation, it is plausible that subsequent loneliness lowered the threshold of requesting help. Moreover, in line with the current literature, a relationship between loneliness and problem gambling has been established (36–38), with loneliness triggering the desire to gamble (39). Media coverage mid-pandemic might also have contributed, emphasising mental health and encouraging help-seeking. Furthermore, a correlation between worsening mental health and COVID-19 has been found (40), possibly making gambling problems appear less manageable due to mental challenges. Lastly, increased help-seeking could simply be attributed to a rising need for support.

Theme two captured obstacles in offering support. A systematic review and meta-analysis illustrated the impact of such obstacles, finding that only 1 in 25 moderate-risk gamblers and 1 in 5 people with problem gambling have sought help (41). Additionally, shame might contribute to such obstacles. Gambling addiction is indeed associated with shame and guilt (42), hindering admission of problems and impeding help-seeking/acceptance. Trivialization of problems, especially in the context of the severe pandemic, might also have contributed. Fewer gamblers seeking/accepting help could additionally be attributable to a lesser need or help being sought elsewhere. Declining phone calls might also reflect a trend in the general population and do not necessarily indicate rejection of support. Lastly, proactive calls may have been perceived as measures of control or threats to the gambler’s autonomy, making it a rejection of control rather than support.

Theme three illustrated how COVID-19 presented an opportunity for change, with many growing aware of their problems. This awareness is likely related to acceptance and action, whereas denial, on the other hand, might be associated with refusing help. Interestingly, the effect of awareness did not persist for all, as some resumed gambling once possible. This highlights availability’s strong influence - a notion supported by Mann (43), which establishes availability as a general risk factor for addictions. Moreover, the theme is supported by a Finnish study revealing that reduced gambling availability during COVID-19 lessened the total consumption and reduced the risk of gambling-related harm (44).

Theme four highlighted gambling’s allure. The heavy advertising described connects to research illustrating its influence on gambling behaviours (45–47). Moreover, the theme illuminated a subgroup of gamblers: escape gamblers (48). From the results, social isolation may potentially initiate/escalate gambling issues, increasing the urge to escape-gamble. This finding coincides with a study conducted during COVID-19, showing a correlation between excessive gambling and escapism (49). Furthermore, even though most land-based gambling opportunities closed periodically during COVID-19, theme four captured the perceived increase in gambling’s availability. This is likely related to online gambling and should primarily have applied to gamblers without previous experience of online gambling. Interestingly, a Norwegian population survey reported an increase in individuals gambling online from 2019 to 2022 (from 58.0% to 65.8%) (11). However, other contributing factors were highlighted, such as finances, marketing, and altered routines.

Lastly, theme five portrayed COVID-19 as having minimal impact on support services and gamblers. The participant who most clearly conveyed this message was a proactive caller – participant 6. Proactive calls may have rendered different responses in contrast to client-initiated contact. It is conceivable that self-referring clients are more motivated to talk and receive help, whereas recipients of proactive calls might be unprepared, possibly providing shorter answers and refusing problems. One study on barriers to help-seeking for problematic gambling found that pride, shame, and denial were most frequently reported as actual or perceived obstacles (50). Such emotional responses may have led gamblers receiving proactive calls to downplay or deny problems, or to respond briefly and defensively, which in turn may have given proactive callers an impression of COVID-19 as unimpactful. Moreover, the study found that non-help-seekers endorsed such possible barriers more often than help-seekers, highlighting the distinction between the groups. Nevertheless, not all proactive callers supported the perception of insignificant impact, adding complexity to the differing perspectives. Given the small number of proactive workers participating in this study (n = 3), it would have been valuable to examine whether a larger sample could clarify if these differences reflect meaningful variation or simply individual idiosyncrasies. In addition, two workers at self-directed helplines also contributed to this theme, complicating the notion of differing response-styles of self-referring clients and recipients of proactive calls. However, one of these individuals was not engaged in helpline operations during the pre-pandemic period, which limits the person’s experience and basis for comparison. This should be considered when interpreting their contribution. It is possible that discrepancies resulted from coincidences or that they were linked to the helper’s personal attributes (e.g., speaking style or approach). Most participants agreed that COVID-19 affected gambling and gambling-related conversations. Notably, proactive callers continued contacting gamblers with the same indications as pre-outbreak, namely at-risk customers. This implies few changes in their work. However, it should have been possible to detect changes in the number of at-risk customers contacted, a notion which, for instance, was refused by Participant 6. Lastly, gambling may have continued unchanged, hence the perception of insignificant impact, whereas help-seeking increased due to social isolation, financial difficulties, and/or family proximity.

Considering our results as a whole, certain findings were consistent with prior research. The subtheme “Augmented awareness of issues affecting oneself or a significant other” suggested that increased awareness of gambling issues led players to address their problems. However, only one participant explicitly suggested decreasing gambling activity during COVID-19. Some stated that people who gambled less were unlikely to contact helplines, meaning helplines had little firsthand experience with declining gambling activity. Yet, participants provided feasible explanations of decreased gambling activity, supporting the findings of Sachdeva et al. (21), implying that some experienced fewer gambling problems during COVID-19, whereas others’ problems worsened. Additionally, aligned with our findings, Turner et al. (25) found that increased marketing and expanded gambling opportunities later in the pandemic led to rising helpline calls. Numbers from Spillavhengighet Norge support the notion of rising inquiries during COVID-19 (33). Lastly, Turner et al. (23) implied a younger audience post-outbreak, also consistent with our results.

The remaining findings challenge previous research, where most studies found a decrease in overall gambling activity during COVID-19. However, research in this field is limited, and the methodologies used in previous epidemiological studies differed from the present qualitative study. Moreover, this study is the first to examine the perspective of help providers during COVID-19, which may explain the contrasting findings. Lastly, the Norwegian context must be taken into consideration, possibly contributing to discrepancies.

Importantly, in addition to lending support to as well as contrasting previous findings, this study makes a unique contribution to the field in several ways. First, the present study advances the existing knowledge by capturing the novel perspective of help providers, illustrating how practitioners also observed qualitative changes in gamblers, gambling habits and help-seeking behaviours during the pandemic. Second, our study offers insights derived from the Norwegian context, offering a point of comparison for international settings. Finally, our qualitative findings add nuance and depth to the quantitative trends observed in prior studies, illustrating that gambling habits and support-seeking behaviours were complexly impacted by COVID-19. As such, our study complements, contributes to and advances the current literature on gambling and gambling support service provision during times of crisis.

## Limitations

The study has several limitations. First, we occasionally observed a strong consensus between participants from the same organizations. This may be due to shared experiences or pre-interview discussions. While some indications were suggesting the latter, these conversations may also have occurred naturally, rather than being deliberate actions. It is also possible that co-workers encounter similar clientele. Nevertheless, it is impossible to determine the exact reasons behind the agreements. Second, the significant differences between the two subgroups of gamblers discussed (self-referring vs. proactively contacted) must be taken into account, as these distinctions may obscure a clear interpretation of the findings and contribute to the observed inconsistencies. Self-referring individuals actively seek help, whereas proactively contacted gamblers rarely exhibit ongoing help-seeking behaviours. We acknowledge that including both proactive help providers and workers at self-directed helplinesin the sample creates the potential for variability in themes and subthemes, as some experiences may be specific to one service type. In practice, the perspectives of the two groups were largely similar across all themes, with some differences concerning the final theme only. Nevertheless, we consider the combined sample valuable in reflecting the whole spectrum of help provision, offering richer and more nuanced findings than what would be achieved by studying the different service types separately. Third, generalisations require caution. Because the sample consists of help providers - who, due to their work, primarily interact with struggling gamblers and are therefore unlikely to report declining gambling problems - caution should be exercised when generalising the findings to problem gamblers as a whole. Additionally, while our findings offer insight into gambling help provision in Norway, the results may not translate to other populations or cultural settings. Differences in health care systems, organizational structures, and cultural attitudes across countries may limit direct generalizability. Further research is thus required to investigate whether similar patterns occur in different nations or with other forms of support services. Fourth, some areas of focus were largely quantitative, which the study’s sample was not ideally suited to address. Nevertheless, they were included due to their importance to participants. A final limitation concerns the potential for bias, as participants may have been prone to favourably depicting their organisation. Moreover, as interviews were conducted in 2024, several years after the pandemic, there is also the potential of recall bias.

## Implications

Despite the limitations, the present study offers novel findings on the experiences of gambling help providers during COVID-19 and their insights into gambling behaviour. First, the results emphasize the importance of prioritizing and maintaining gambling support services during times of crisis. Second, the findings suggest that stakeholders should recognise how social isolation may heighten the urge for escape, thereby increasing the risk of gambling. In the event of future crises, recommendations and prevention programs should be mindful of these effects, making sure to attend to especially vulnerable groups. Third, the findings indicate that social isolation within the family unit may reduce openness and help-seeking/acceptance. Accordingly, action should be taken to facilitate communication with help providers. Examples of such measures include flexible opening hours and chat services. Fourth, the findings encourage the regulation of online gambling in times of social isolation. Increased frequency of enforced breaks, reduced opening hours, and lowered loss limits may prove beneficial. Fifth, the findings suggest that increasing the visibility of support services may be advantageous. Sixth, our findings demonstrate how diverse individuals may respond to crises and lockdowns, emphasizing the need for adaptable interventions. While some individuals may require increased access to support during major societal disruptions, others may benefit from alternative leisure activities to help them cope with restrictions and to structure their time (51). Taken together, the insight provided in the present paper may guide responses and interventions during future comparable events.

## Conclusion

This study investigated how Norwegian gambling help providers experienced COVID-19’s impact on gambling and gambling-related conversations, offering insights into both the help provider experience and problem gambling as experienced by said workers. The findings suggested varied experiences, likely affected by affiliated organisations and the directionality of calls. However, all themes but one indicated substantial shifts throughout COVID-19, reflected in gambling behaviours and conversations. Both increased help-seeking and barriers in offering support were reported. Additionally, both gamblers’ addressing their problems and gambling being especially enticing emerged as frequent experiences. The findings offer valuable and novel insights applicable to future crises. We encourage researchers to further replicate and supplement these findings. Interview-based studies investigating how gamblers or affected others experienced COVID-19, including potential long-term or delayed effects, could yield valuable supplemental results.

## Data Availability

The raw data supporting the conclusions of this article will be made available by the authors, without undue reservation.
