# Supplementary material for: Help-seeking for gambling problems during the COVID-19 pandemic: a qualitative study on the experiences of Norwegian help providers and their insights into gamblers
Source: Front Psychiatry. 2026 Feb 27;17:1734445. doi: 10.3389/fpsyt.2026.1734445 (PMC12982926; doi:10.3389/fpsyt.2026.1734445)
Supplement: Supplementary file 1 [file Table1.docx]

*Interview Guide*

**Interview guide (semi-structured)**

**Background information about the participant**

- Age?
- Gender?
- What is your role in connection with the helpline/proactive conversations?
- How long have you been in this role?
- Approximately how many conversations have you had in this role?

**Impact of the COVID-19 pandemic**

How did you experience the COVID-19 pandemic’s effect on the content of the conversations?

Can you provide a specific example of a conversation where the pandemic significantly affected the caller?

How did you experience the COVID-19 pandemic’s impact on the number of conversations?

What topics related to the COVID-19 pandemic came up in conversations?

Was there anything about the conversations during COVID-19 that was different compared to the period before?

Was there anything about the conversations during COVID-19 that was different compared to the period after?

Did you perceive that the various phases of the pandemic (such as lockdown and reopening) affected the conversations, and if so, how?

In certain periods, some gambling options were closed due to the pandemic. How did you experience this affecting the conversations?

Regarding gambling problems – what (if anything) did you experience as positive about the COVID-19 pandemic?

Regarding gambling problems – what (if anything) did you experience as negative about the COVID-19 pandemic?

Have you observed any lasting changes (e.g., in callers’ gambling behaviour or problems) after the COVID-19 pandemic? If yes, what?

If the COVID-19 pandemic caused some people to gamble more/spend more money – what do you believe are the reasons/mechanisms behind this?

If the COVID-19 pandemic caused some people to gamble less/spend less money – what do you believe are the reasons/mechanisms behind this?

**Interview closing**

How was it for you to talk about this topic?

Is there anything we haven’t discussed that you believe is relevant or interesting for our understanding of the topic?

What do you think we should focus on in future research concerning those working on helplines for gambling problems (what topics are important to you)?
